# COVID-19 and dental sleep medicine: risks, precautions and patient guidance

**DOI:** 10.5935/1984-0063.20190156

**Published:** 2020

**Authors:** Thays Crosara Abrahão Cunha, Rowdley Rossi, Lilian Chrystiane Giannasi Marson, Thais M Guimaraes, Marco Antônio Cardoso Machado, Miguel Meira-e-Cruz, Otávio Ferraz, Milton Maluly Filho, Paulo Afonso Cunali, Cauby Maia Chaves Júnior, Cibele Dal-Fabbro

**Affiliations:** 1 Universidade Federal de Uberlândia, Instituto de Biotecnologia - Uberlândia - Minas Gerais - Brazil.; 2 Universidade Federal de São Paulo, Departamento de Pneumologia - Sao Paulo - Brazil.; 3 UNESP - SJC, Biociências - São José dos Campos - Sao Paulo - Brazil.; 4 UNIFESP - Escola Paulista de Medicina, Departamento de Psicobiologia - Sao Paulo - Brazil.; 5 UNIFESP - Escola Paulista de Medicina, Departamento de Neurologia - Sao Paulo - Brazil.; 6 University of Lisbon, Sleep Unit - Cardiovascular Center - Lisbon - Portugal.; 7 Instituto do Sono, Dentistry Division - Sao Paulo - Brazil.; 8 Hospital Marcelino Champagnat, Corpo Clínico - Curitiba - Paraná - Brazil.; 9 Federal University of Ceará, Division of Orthodontics and Pediatric Dentistry - Fortaleza - Ceará - Brazil.

**Keywords:** Sleep, Dentistry, Coronavirus Infections, Sleep Apnea, Obstructive

## Abstract

COVID-19 is the offcial name for the disease caused by SARS-CoV-2, which has become a pandemic, infecting more than 5 million people worldwide. Transmission occurs by inhaling droplets generated when an infected person coughs, sneezes or exhales, or by touching contaminated surfaces and then rubbing their hands over their eyes, nose or mouth. Some infected people become seriously ill, while others have no symptoms, but even though they are asymptomatic, they can still transmit the virus. As vaccines and effective medications do not yet exist, the only way to handle the devastating consequences of the pandemic is prevention. Quality of sleep is essential for the immune system to be prepared to receive, ﬁght and restore itself after a viral infection. Therefore, patients with obstructive sleep apnea (OSA) should continue treatment, and only suspend or change the therapeutic modality under the guidance of a sleep physician. In the era of COVID-19, due to the high probability of contamination promoted by CPAP, the mandibular repositioning device has been considered as the ﬁrst choice for patients with OSA. However, as the dental approach is at high risk of contamination, due to the proximity of the dental surgeon to the patient, it is essential that the professional who works in this ﬁeld knows the risks to which they are exposed. Precautions must be adopted and patients should be guided in order to control and use of their intraoral devices.

COVID-19 is a respiratory disease that has become a pandemic, caused by the SARS-CoV-2 virus^^1^^. Some infected people become seriously ill, while others have no symptoms, but even though they are asymptomatic they can still transmit the virus^^2^,^3^^.

The literature describes that the state of sleep deprivation or insufficient sleep increases the potential for rhinovirus infection^4^ and decreases the magnitude of the immune response to the hepatitis B virus^^5^^. Among others, these studies point to the regulatory role that sleep plays on immune functions^^6^^. Knowing the importance of sleep for the proper functioning of the immune system, patients with obstructive sleep apnea (OSA) should not suspend or alter their treatment without specialized medical recommendation.

OSA is a sleep-related respiratory disorder characterized by recurrent respiratory arrest leading to intermittent hypoxia and awakening while the patient sleeps^^7^^, featuring a state of chronic sleep deprivation. The gold standard of treatment for OSA is CPAP (continuous positive airway pressure). This device generates and directs a continuous flow of air through a mask firmly adhered to the patient’s face, promoting unblocking of the upper airways (VAS)^^8^^. In COVID-19 times, there is a special concern with the users of these devices, due to the potential escape of air causing the spread of the virus if the patient is infected, symptomatic or not^^9^,^10^^. In cases of patients with COVID-19 at home, they should sleep in isolation. However, the concern is in hospital or community settings, such as a nursing home, where the possibility of contamination is exponential^^11^^.

Oral appliances constitute another form of treatment, particularly the customized mandibular advancement devices (OA_MAD_). This device stabilizes the mandible in a more anterior position, promoting unblocking of the upper airways^^12^^. OA_MAD_ is routinely indicated for milder cases of OSA. The preparation, installation and adjustment is performed by a dentist with training in sleep dentistry, and must meet the indications of a sleep physician. Due to the high probability of contamination promoted by CPAP, OA_MAD_ has been considered as the first choice for patients with OSA and COVID-19^^13^^. Since, in addition to not producing contamination of the environment by air leakage, it keeps the patient with his mouth closed, which reduces the spread of salivary particles, in addition to being proven effective in the treatment of OSA^^13^^.

Given the seriousness of the COVID-19 pandemic, it is important that health professionals follow the guidelines of the health regulatory agencies in their countries. However, these guidelines are generic and may not address risks unique to specific specialties. Determining a better way to prevent transmission is now the key to reducing the spread of the virus. Therefore, we present below, clarifications and guidelines for dentists working in dental sleep medicine.

## What are the risks of COVID-19 for the practice in sleep dentistry?

The SARS-CoV-2 virus uses the ACE2 receptor (angiotensin-converting enzyme 2) from the membrane-bound host to penetrate cell, being found mainly in mucosal tissues, such as the back of the tongue and salivary glands^^14^,^15^^. Saliva is considered a reservoir of the virus, a vehicle for the spread of the disease and is suggested as a possible fluid for noninvasive diagnosis of COVID-19^^16^,^17^^.

Respiratory droplets excreted from the oral cavity, during speech, for example, reach up to 1.5 meters away. In case of coughing and sneezing, they can reach up to 3 meters^^18^^. As the dentist is about 60 centimeters or less from the patient’s oral cavity, he is prone to receiving a high viral load during the procedure in a possible carrier of COVID-19, regardless of the intervention he performs^^19^^.

Even more importantly, studies show that after using the dental aerosol (high speed drill with water spray), the particles are suspended in the air for up to 30 minutes^^20^,^21^^. These particles are deposited on any horizontal surface, remaining active for up to 7 days on plastic surfaces^^22^^.

## What precautions should be taken?

As the individual can transmit the virus without showing any clinical symptoms, all patients in a dental environment should be considered as a potential carrier of COVID-19.

So far, infection control measures are the only option to minimize the spread of the disease, since vaccines and drugs are not yet available^^23^^. Some precautions can be taken to minimize contamination/spread in the dental environment^^24^^:

Before face-to-face assistance, it is recommended:

- The initial demand for assistance to be carried out by telephone, e-mail and/or video conference. This can help in the primary management of emergencies, avoiding face-to- face assistance;
- People with a confirmed diagnosis, who report signs and symptoms, or who have been in direct contact with confirmed cases of COVID-19, should not attend the dental clinic;
- Use of digital oximeter and forehead thermometer are recommended as soon the patient arrive at the dental clinic;
- Minimum distance of 1.5 meters between patients in the waiting room;
- Surfaces must be disinfected before and immediately after dental care, applying for at least 62% alcohol, 0.5% hydrogen peroxide or 1,000 ppm (0.1%) sodium hypochlorite for 1 minute^^25^^;
- Interval of at least 30 minutes between patients;
- Air conditioning and closed windows should be avoided. Natural ventilation is recommended;
- In order to minimize the possible viral load present in saliva, rinse with chlorhexidine gluconate solution (0.12%, 15mL) for 30 seconds. However, as we know, the viral load present in the throat, nose, tongue and saliva are high, and the possibility of contamination in a short space of time are great. It is important to note that, according to Yoon et al.^^26^^, the mouthwash with chlorhexidine supports an effective reduction of viral load for up to 2 hours after this procedure^^14^,^15^,^27^^.

During dental care, it is recommended:

- Protective equipment must protect both the dentist and the patient from possible contagion. It is recommended to use masks with filtration (FFP-2, N95, KN95) and without expiration valves;
- Use of goggles and/or face shield to avoid contagion through the lining of the eyes;
- Wear a hair cap and long sleeve disposable lab coat;
- Molding procedures should be avoided as they encourage coughing and vomiting, so oral scanning, when possible, is recommended^^28^^.

Guidelines for patients with OSA using OA_MAD_:

- Do not interrupt or modify the treatment without supervision by your dentist and/or sleep doctor;
- Inform your dentist and/or sleep doctor if you are diagnosed with COVID-19;
- In case of contamination by COVID-19, isolation and not sharing the room with other people is recommended;
- Because it is in close contact with saliva, OA_MAD_ is considered a reservoir for the virus and therefore it must be cleaned with great care, with special attention to retentive areas;
- it is important to mention that no solid evidence on most powerful, read balance efficacy and safety, on best substance to be used to clean the OA_MAD_^^29^^, this way we will recommend the same guidelines as indicated for CPAP equipment:
  - *Before inserting the OAMAD into the dental arch and after removing it the following day:*
    - Hand hygiene with water and soap;
    - Emerge the device toothbrush used to clean the appliance in a detergent and water solution for 30 minutes. When possible, submerge in enzymatic detergent for 5 minutes. Brushing OA_MAD_ with detergent/enzymatic detergent also is important;
    - For devices that do not have metallic components, the immersion can be carried out in 2.5% sodium hypochlorite solution for 5 minutes;
    - For both cleaning ways, finalize rinsing with plenty of running water.

We understand that we are living in an acute moment of dissemination of COVID-19, and the soon we will reach a standard of normalization by resuming daily clinical activities. As we decrease the distance and social isolation, we increase the possibility of contagion, therefore, it is essential that dental sleep doctors be prepared to review and guide their patients.
